# Assessing physical activity/behavior of adolescents living in the Pacific with accelerometer data: 231 GENEActiv records in New Caledonia

**DOI:** 10.1016/j.dib.2024.111228

**Published:** 2024-12-15

**Authors:** Guillaume Wattelez, Stéphane Frayon, Olivier Galy

**Affiliations:** a Service Unit, University of New Caledonia, Noumea, New Caledonia; b Interdisciplinary Laboratory for Research in Education, EA7483, University of New Caledonia, Noumea, New Caledonia

**Keywords:** Physical movement, Accelerometry, Objective measure, Signal vector magnitude, Lifestyle, Teenagers, Pacific Island countries and territories, Melanesia

## Abstract

This paper presents a dataset related to the physical activity behavior of 206 adolescents (107 females and 99 males) from 11 to 16 years old and 25 adults (13 females and 12 males) living in rural (77 adolescents and 15 adults) and urban (129 adolescents and 10 adults) parts of New Caledonia, an archipelago of the South Pacific. Physical behavior was assessed through 60-Hz triaxial GENEActiv accelerometers worn for 5 to 7 consecutive days between July 2018 and April 2019. Participants were randomly recruited at school and trained staff fitted the devices on the nondominant wrist, at which time all were reminded of the expectations while wearing the device.

The dataset contains restricted raw .bin accelerometer data provided by the GENEActiv software and open 1-second epoch .csv accelerometer data derived from the .bin files with an R script, provided in the repositories as well. Similarly, there is open information (age range and sex) and restricted information (age in months, place of living, declared cultural community and socioeconomic status) about the participants.

The paper describes the file contents, describes the participants involved when collecting this dataset, and gives examples of how the data can be processed to assess the physical behavior of these participants.

This high-quality dataset contains diverse sociodemographic information about the participants, which will enable the determination of correlations with their physical behavior. Moreover, since raw data with high granularity and continuous information is provided, studies interested in data processing and testing new methods on time series analyses can use the dataset.

Specifications Table

**Table:** 

Subject	Public health and health policy
Specific subject area	Assessing the physical activity behavior of adolescents living in the Pacific, especially in New Caledonia
Type of data	Raw (.bin and .csv files), Description table (.csv file).
Data collection	Data were recorded with a 60-Hz triaxial accelerometer (GENEActiv; Activinsights Ltd, Kimbolton, UK) for 5 to 7 consecutive days, ranging from July 2018 to April 2019. Trained staff put the device on the participants’ nondominant wrist. Participants were recruited according to the school where they were enrolled [1,2]. Eight secondary public schools were selected based on their location (i.e., rural/urban and New Caledonia Province), size (N>200 students) and school staff agreement; adolescents were then randomly selected to wear the device. Some parents of the selected adolescents were also asked to wear the device. Written consent was obtained from the adolescents and their parents before the study.
Data source location	Country: New Caledonia Towns/Villages: Dumbéa, Koumac, Lifou, Nouméa, Païta, Poindimié
Data accessibility	Repository name: Zenodo Data identification number: Accelerometer part 1: 10.5281/zenodo.11594645 (restricted) Accelerometer part 2: 10.5281/zenodo.12638965 (restricted) Accelerometer part 3: 10.5281/zenodo.12661429 (restricted) Additional information: 10.5281/zenodo.12195186 (restricted) Accelerometer part 1: 10.5281/zenodo.12615468 (open) Accelerometer part 2: 10.5281/zenodo.12638746 (open) Accelerometer part 3: 10.5281/zenodo.12682660 (open) Direct URL to data: Accelerometer part 1: https://doi.org/10.5281/zenodo.11594645 (restricted) Accelerometer part 2: https://doi.org/10.5281/zenodo.12638965 (restricted) Accelerometer part 3: https://doi.org/10.5281/zenodo.12661429 (restricted) Additional information: https://doi.org/10.5281/zenodo.12195186 (restricted) Accelerometer part 1: https://doi.org/10.5281/zenodo.12615468 (open) Accelerometer part 2: https://doi.org/10.5281/zenodo.12638746 (open) Accelerometer part 3: https://doi.org/10.5281/zenodo.12682660 (open)
	Instructions for accessing these data: An embargo has been applied on the public data that will be freely and openly available under the CC-BY 4.0 licence on 31 December 2025. Each public link gives access to a part of the raw .csv accelerometer data files and to a .csv file describing participants’ de-identified information. Restricted data, i.e., raw .bin accelerometer data files and detailed information about participants, can be accessed on demand by leaving a message with the Zenodo user system management. Each serious request will be examined. Although specific conditions of access may be specified according to the studies and treatments envisaged, the generic Data Use Agreement is in supplementary materials. The minimum requirement in terms of ethics is General Data Protection Regulation (GDPR) compliance and no re-identification process. The team providing the restricted data also expects collaboration or acknowledgment (to be discussed with the demanders).
Related research article	Wattelez, G., Amon, K. L., Forsyth, R., Frayon, S., Nedjar-Guerre, A., Caillaud, C., & Galy, O. (2024). Self-reported and accelerometry measures of sleep components in adolescents living in Pacific Island countries and territories: Exploring the role of sociocultural background. Child: Care, Health and Development, 50(3), e13272. https://doi.org/10.1111/cch.13272

## 1 Value of the Data

• Although available accelerometer datasets are rather scarce, especially in the Pacific, this unique dataset provides objective data, for the first time, on more than 200 participants belonging to an understudied population in the literature, including Melanesian and Polynesian participants. The data are sex-balanced, with proportions similar to what is encountered in New Caledonia, in terms of sociodemography and socioculture.
• These 60-Hz and 1-second epoch data files provide high granularity and a large quantity of continuous information about the participants’ physical behavior over a relatively long period (i.e., from 5 to 7 days), compared with other studies’ records.
• This dataset contains records of real-life movements, naturally captured with noninvasive devices.
• This information can be derived to assess physical activity behavior, sleep behavior or other components related to movement records. By using the simple or the additional information, correlations may be highlighted between sociodemographic or sociocultural features and movement behavior.
• Movement can be further analyzed using artificial intelligence models to detect specific patterns or, conversely, this dataset can be used to improve artificial intelligence models related to time series.
• As these are raw data, researchers will be able to use and process the data themselves with new future methods.

## 2 Background

Since the 19th century, Pacific Island Countries and Territories (PICTs), including New Caledonia, have undergone a profound socioeconomic transition that has affected both physical activity and dietary patterns and, more generally, the lifestyle of the various populations. The Second World War in particular had a substantial impact on these populations, with an increase in the availability of industrialized processed food and the spread of cheap and low-cost products. Urban migration led many to partially set aside traditional means of subsistence based mainly on farming, fishing and hunting in favor of paid work [3,4]. These changes induced a shift not only in diet, but also in physical activity behaviors, resulting in a pandemic obesity crisis.

We focused especially on the physical behavior of adolescents living in PICTs, over 30 % of whom are overweight or obese [2,5]. For this purpose, we collected objective data to assess their behavior related to or assessed by movement [6,7].

## 3 Data Description

### 3.1 Repository and file description

Due to the large size of the files and the size limitations of repositories (50 Go max and 100 files max per dataset), this dataset consists of 3 open repositories with accelerometer .csv files, 3 restricted repositories containing accelerometer .bin files, and one restricted repository with a file containing sensitive information. Table 1 provides information about the sizes of the repositories. The anonymized versions are open whereas the non-anonymized versions are restricted.

**Table 1:** List of the repositories included in the dataset and their information: DOI, number of files contained, file size (in Go).

Repository title	Repository version/part	DOI	Number of files	File size (in Go)
GENEActiv accelerometer files collected during the project titled “Cultures et comportements alimentaires de la jeunesse dans les pays francophones du Pacifique au XXIème siècle: exemple de la Nouvelle-Calédonie” [Eng: “Eating cultures and behaviors of young people in French-speaking Pacific countries in the 21st century: the example of New Caledonia”]	(non-anonymized version - first part)	10.5281/zenodo.11594645	75	46.85
	(non-anonymized version - second part)	10.5281/zenodo.12638965	76	48.38
	(non-anonymized version - third part)	10.5281/zenodo.12661429	80	48.13
	(anonymized version - first part)	10.5281/zenodo.12615468	77	13.78
	(anonymized version - second part)	10.5281/zenodo.12638746	78	13.29
	(anonymized version - third part)	10.5281/zenodo.12682660	82	14.21
Information associated with GENEActiv accelerometer files collected during the project titled “Cultures et comportements alimentaires de la jeunesse dans les pays francophones du Pacifique au XXIème siècle: exemple de la Nouvelle-Calédonie” [Eng: “Eating cultures and behaviors of young people in French-speaking Pacific countries in the 21st century: the example of New Caledonia”]	(non-anonymized information)	10.5281/zenodo.12195186	1	/

Raw .bin files stored in non-anonymized accelerometer repositories were extracted with the GENEActiv software after the device was removed from the participant's wrist. Open repositories contain accelerometer .csv files extracted from .bin files with the R library GENEAread and saved with a 1-second epoch [8,9]. Such a repository also contains a file named “read_a_binFile_share.R” that shows an example of R code used to read a .bin GENEActiv file, convert raw data to 1-second epoch data, and save the output in a .csv file.

Each repository dedicated to accelerometer files also contains a file named “participantCharacteristics.csv” that provides basic information about each participant and the record of the full dataset (Table 2). We were careful to list all the files available from the current study, whatever the repository, to ensure that it would be easy to download all the data when accessing one repository.

**Table 2:** Description of the content of participantCharacteristics.csv and the participantCharacteristics_nonAnonymized.csv files.

Column name	Description	Availability
Participant ID	Participant ID for this study	Open
Participant status	Participant status in the study (“Adolescent” or “Adult”)	Open
Age in months	Age in months of the adolescent participant; unknown for adults	Restricted
Age in years	Age in years of the adolescent participant; unknown for adults	Restricted
Age range	Age range of the adolescent participant (“Less than or equal to 12”, “Between 13 and 14 inclusive” or “Greater than or equal to 15”); unknown for adults	Open
Sex	Sex of the participant (“Female” or “Male”)	Open
Place of living	Place of living according to school location (“Rural” or “Urban”)	Restricted
Cultural community	Declared cultural community (“African,” “Asian,” “European born in France or elsewhere,” “European Caledonian (born in New Caledonia),” “Melanesian,” “Polynesian” or “Other”)	Restricted
International SES	International socioeconomic status (“High,” “Medium” or “Low”)	Restricted
GENEActiv file	GENEActiv file (with no extension) associated to the participant	Open
Starting day	Starting day of data collection, i.e., first day the device was worn	Open
Day of removal	Day of device removal, i.e., last day the device was worn	Open
DOI of the non-anonymized repository	DOI of the repository where the .bin file is stored	Open
DOI of the anonymized repository	DOI of the repository where the .csv file is stored	Open

The restricted repository dedicated to participant information contains only a non-anonymized version of the participant characteristics, stored in a file named “participantCharacteristics_nonAnonymized.csv.” This file is structured in the same way as the participantCharacteristics.csv files, but with additional columns also listed in Table 2 and noted “Restricted” in the Availability column. Although there is no name in this dataset, we cannot widely spread this information, which is considered to be potentially sensitive personal data.

An accelerometer .bin file can be processed with open libraries, like GENEAread or GGIR for R [[10], [11], [12], [13]]. It can also be processed with the free GENEActiv software for a .csv conversion [https://activinsights.com/support/geneactiv-support/ accessed on 2024–06–27]. Table 3 describes the data contained in a .bin file.

**Table 3:** Overview of data contained in an accelerometer .bin file*.

Information	Description
timestamp	Time of measurement
x	x axis acceleration (g)
y	y axis acceleration (g)
z	z axis acceleration (g)
light	Light level (lux)
button	Button pressed (0 or 1): no importance in our case, as our devices have no buttons
temperature	Recorded temperature (°C)

⁎ Information collected in [14] and [15].

The accelerometer .bin files also contain metadata about the record, including the device's unique serial code, measurement frequency, measurement period and start time. Metadata also contains information related to the participant (e.g., birthdate). For this reason, .bin files are not openly available, even though this information may be erroneous in our files.

An accelerometer .csv file is the output of a raw .bin read with the GENEAread R library and processed with an R script aimed at aggregating the 60-Hz raw data into 1-second epoch data, deriving the information to compute signal vector magnitude (SVMg), and saving the output into a .csv file [9,10]. Table 4 describes data stored in an accelerometer .csv file.

**Table 4:** Overview of data contained in an accelerometer .csv file*.

Column index	Information	Description
1	timestamp	Time of measurement at the end of the epoch
2	xm	Mean x axis acceleration (g)
3	ym	Mean y axis acceleration (g)
4	zm	Mean z axis acceleration (g)
5	lightm	Mean light level (lux)
6	tempm	Mean temperature (°C)
7	svmgsum	Sum of vector magnitude
8	sdx	Standard deviation x axis acceleration (g)
9	sdy	Standard deviation y axis acceleration (g)
10	sdz	Standard deviation y axis acceleration (g)
11	id	Name of the .bin file (not available in files generated by the GENEActiv software)

⁎ Some information was collected in [14] and [15].

### 3.2 Description of the participants

Table 5 describes the participants of this dataset. In both adolescents and adults, about 52 % were females. In adolescents, 37 % lived in a rural environment, whereas 60 % of the adults did. In adolescents, almost a third were 12 years old or younger and almost half were between 13 and 14 years old; the others were 15 years old or more. The average age of the adolescent participants was 13.1 (sd = 1.25) but we do not have age information for adults.

**Table 5:** Distribution of sex, place of living in adolescents and adults, and age range in adolescents.

		Adolescents [N (%)]	Adults [N (%)]
Sex	Female	107 (51.9)	13 (52.0)
	Male	99 (48.1)	12 (48.0)
Place of living	Rural	77 (37.4)	15 (60.0)
	Urban	129 (62.6)	10 (40.0)
Age range	Less than or equal to 12	71 (34.5)	/
	Between 13 and 14 inclusive	98 (47.6)	/
	Greater than or equal to 15	37 (18.0)	/

Regarding the restricted information, the socioeconomic status was known for all the adolescent participants but the cultural community was known for only 198 of them.

### 3.3 Example of an accelerometer series

**Fig. 1:**
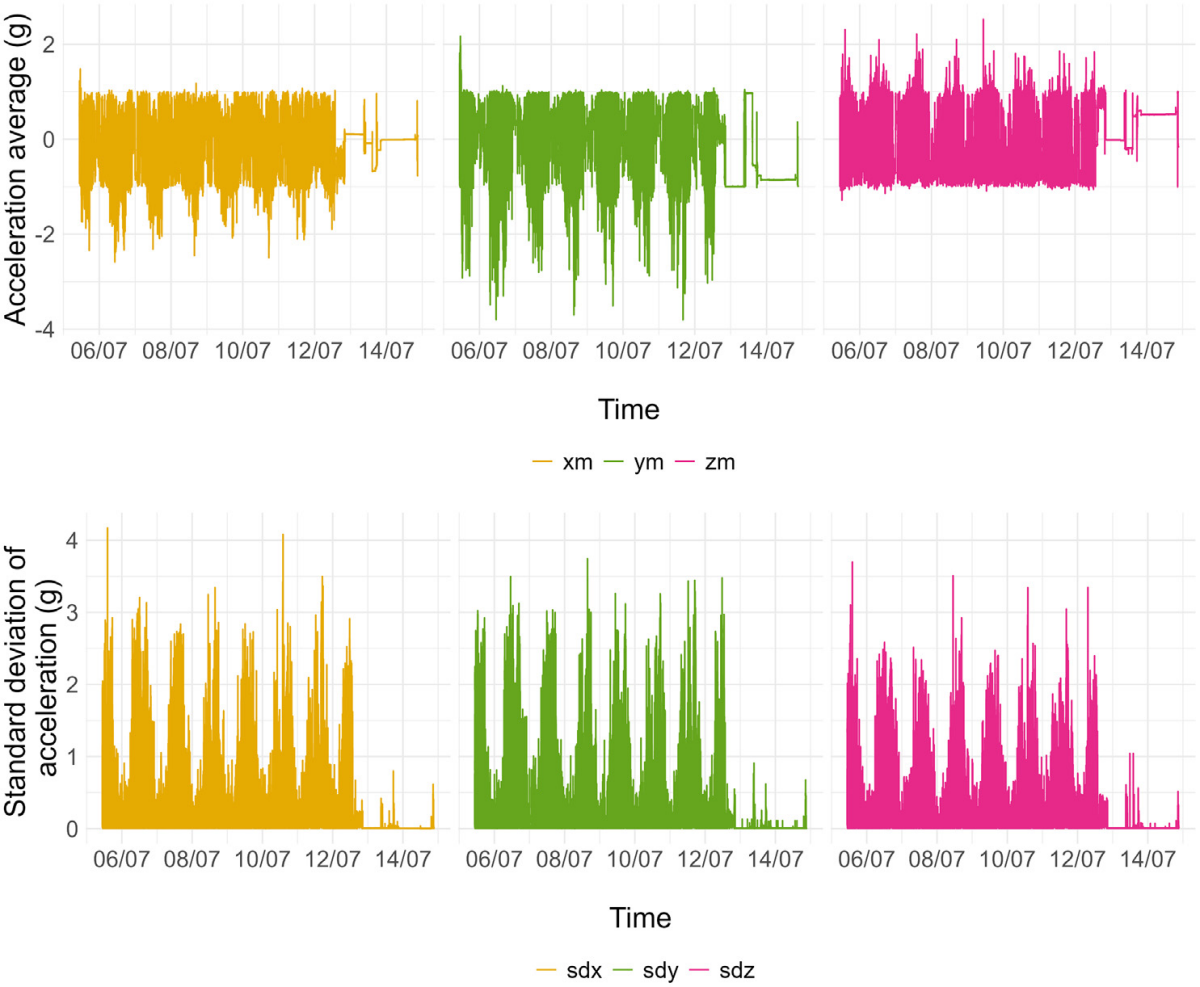
Graphs of data contained in a .csv accelerometer file: acceleration average in 1-second epochs in x (yellow), y (green) and z (pink) axes, and acceleration standard deviation in 1-second epochs in x (yellow), y (green) and z (pink) axes.

**Fig. 2:**
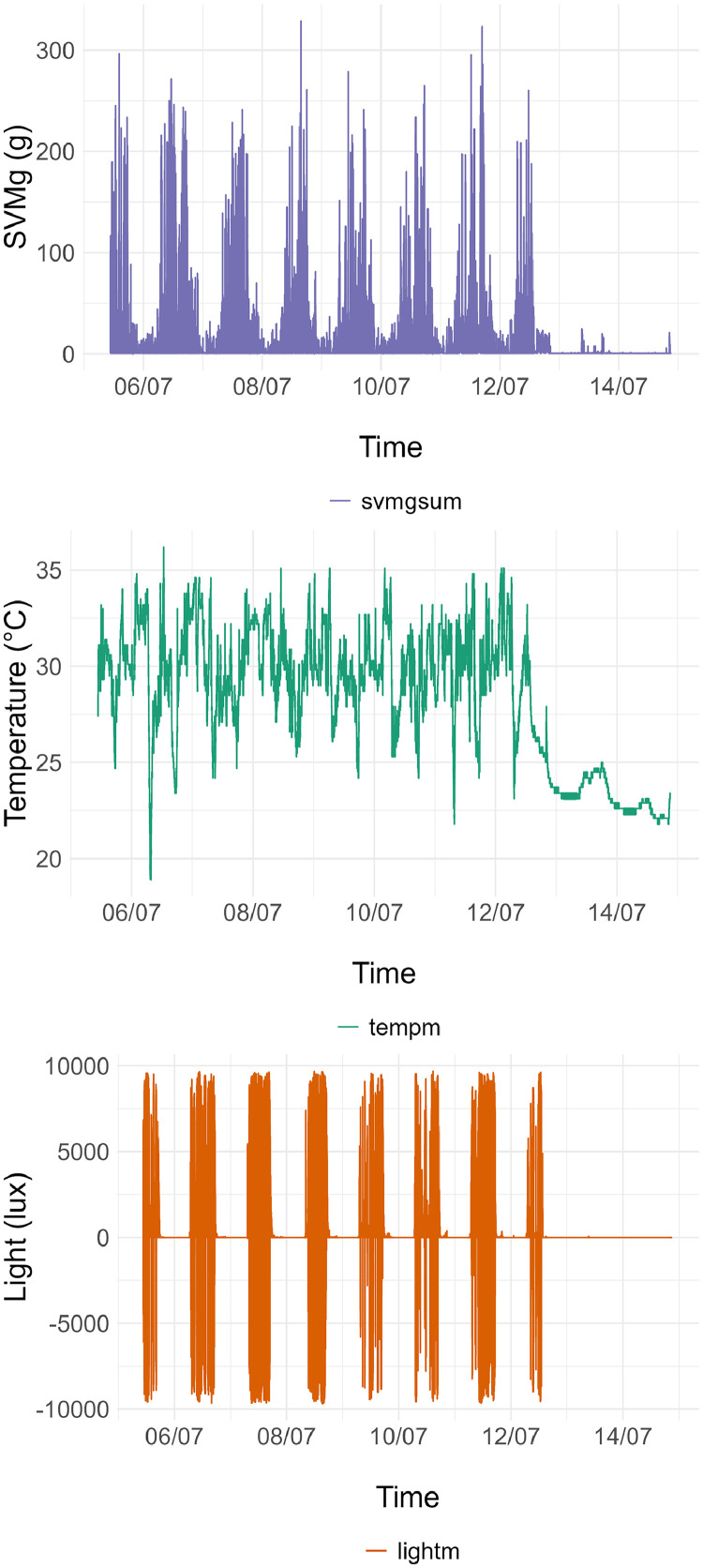
Graphs of data contained in a .csv accelerometer file: signal vector magnitude (purple), temperature (green) and light (orange) in 1-second epochs.

Figs. 1 and 2 are graphs drawn from the .csv file containing data recorded with a device worn by an adolescent participant from 5 to 13 July 2018. Fig. 1 is a representation of 1-second epoch acceleration data (x, y and z axes). The upper graphs are 1-second epoch acceleration averages and the lower graphs are 1-second epoch acceleration standard deviations.

Fig. 2 is a representation of 1-second epoch SVMg, temperature and light data.

Although the device was worn from 5 July to 13 July, the time series does not stop on the day of removal as the data was not extracted before 14 July.

## 4 Experimental Design, Materials and Methods

### 4.1 Recruitment of participants

The current data were collected as part of a larger data collection effort involving about 1200 adolescents. Participants attended 8 different schools in New Caledonia: 5 schools in urban areas (Noumea and its suburb) and 3 schools in rural areas (out of Noumea). Schools were selected according to the following criteria: (1) a representative selection of schooled adolescents between rural and urban area (37 % and 63 %, respectively), (2) a distribution in the 3 provinces close to that observed in the adolescent population (70 % in the Southern Province, 20 % in the Northern Province and 10 % in the Loyalty Islands Province), and (3) school size sufficient to get enough data in a single field trip (N > 200). The schools meeting these criteria were randomly selected and the school staff were contacted to obtain agreement. A staff member randomly selected 6 classes from 6th to 3rd in the French education system, i.e., from 6th grade to 9th grade in the Anglo education system, which left us expecting around 150 participants per school.

Among the students involved in this large study, 211 were randomly selected and agreed to wear an accelerometer device for 7 consecutive days.

We contacted 29 parents of the adolescents selected to wear the accelerometer device and asked them to wear the device, as well. Those who accepted wore the device for 7 consecutive days.

### 4.2 Experimental conditions

We used 60-Hz triaxial GENEActiv accelerometers (Activinsights Ltd, Kimbolton, UK). After asking if the participant was right-handed or left-handed, a trained staff-research member fitted the device on each participant's nondominant wrist, on dorsal side. At this time, all participants were reminded to keep the device on their wrists for at least 7 days and to perform their normal everyday activities (sports, physical activity, sleep, nap, etc.) as when they don't wear the device. It was further specified that it would be best to keep it on until the staff came to pick it up. They were also reminded that the device had to be worn throughout the day and night and not removed except for activities where wearing it could present a hazard, for instance doing fighting sports or regarding any security issue. It has been asked to report the periods of device removal (day, hour and duration) but we did not get any return of this nature.

For the adolescents, the accelerometer devices were adjusted and then collected after the 7 days during class time.

For the adults, a meeting was scheduled directly with the interested party to hand over and adjust the device and provide the explanations and recommendations specified above. After the 7 days, another meeting was scheduled to collect the device.

### 4.3 Data processing

After the recording days, the device was collected and data were extracted using the GENEActiv software providing the restricted raw 60-Hz record .bin files (Activinsights Ltd, Kimbolton, UK; https://activinsights.com/support/geneactiv-support/). Since we were interested in 1-second epoch data, we computed the SVMg, aggregated the 60-Hz records into 1-second records, and saved the output in .csv files. SVMg was calculated following Eq. 1 [9,10].

(1) $$SVMg=\sum_{i}\left|\sqrt{x_{i}^{2}+y_{i}^{2}+z_{i}^{2}}-1\right|$$

From the SVMg value, a physical activity (PA) level can be associated to each recorded time period: sedentary (SVMg < 4.5 g), light PA (4.5 g ≤ SVMg < 16.5 g), moderate PA (16.5 g ≤ SVMg < 42 g) and vigorous PA (SVMg ≥ 42 g) [6]. In many studies, moderate PA and vigorous PA are aggregated to get the category moderate and vigorous PA (MVPA) [8,9].

When handling this kind of data, it is common and recommended to fix bouts according to the PA level. Thus, a second associated to a PA level is actually counted as well only if it is in a sufficiently long time period with this PA level, i.e. there are enough neighboring seconds with the same associated PA level. For instance, in Diaz et al. [9] a MVPA second was counted only if it was in an interval with uniquely MVPA seconds for at least 3 seconds.

### 4.4 Data validity

We were unable to meet 2 parents to hand over the device. After the data extraction, we noticed that 5 devices worn by adolescents and 2 devices worn by parents had remained inactive. We therefore obtained 206 data files from adolescents and 25 data files from parents.

### 4.5 Sociodemographic data

Place of living was determined according to school location and is the criterion used by the European standard to assess the degree of urbanization [16]. The participants who attended and the parents having a child who attended one of the 3 rural schools were classified as “Rural” and the others were classified as “Urban.”

From the school system database, we collected the sex and birthdate of the adolescent participants, as well as information about the parents’ professional occupation. We deduced age in months of the adolescents based on their birthdate and the day of the research intervention. Then, age in years and age range (with 3 categories) were trivially calculated.

Each adolescent's socioeconomic status (SES) was determined based on the professional occupation of the household reference person, defined as the householder with the highest income. The National Statistics Socio-Economic classification was used [17] and then 3 categories were derived to get the “International SES” variable: Low for routine and manual occupations, Medium for intermediate occupations, and High for managerial and professional occupations.

Cultural community was determined through an online questionnaire with the following question: “A quelle communauté te sens-tu appartenir ?” that can be translated as: “Which community do you feel you belong to?” Participants could choose answers usually proposed by the Institut de la statistique et des études économiques (ISEE: Institute of Statistics and Economic Studies) census as recommended by the Institut national de la santé et de la recherche médicale (INSERM: the National Institute of Health and Medical Research) report on New Caledonia [18], but they could choose only one cultural group. The available cultural communities were: “Africaine” (“African” in English), “Indonésienne, Vietnamienne ou Asiatique d'autre origine” (“Indonesian, Vietnamese or Asian from another background” in English and written as “Asian” in the dataset), “Kanak ou Mélanésienne” (“Kanak or Melanesian” in English and written as “Melanesian” in the dataset), “Européenne Calédonienne (né(e) en Calédonie)” (“European Caledonian (born in New Caledonia)” in English), “Européenne metro, né(e) en France ou ailleurs” (“European born in France or elsewhere” in English), “Wallisienne, Futunienne, Tahitienne (Polynésienne)” (“Wallisian, Futunian, Tahitian (Polynesian)” in English and written as “Polynesian” in the dataset), and “Autre” (“Other” in English). It is usual to aggregate categories because of small category sizes. For instance, participants who answered “European Caledonian (born in New Caledonia)” or “European born in France or elsewhere” were aggregated into the “European” category or the “Caucasian” category. Participants who answered “African,” “Indonesian, Vietnamese or Asian from another background” or “Other” were aggregated into the “Other” category. Depending on the study and its aims, participants who answered “Wallisian, Futunian, Tahitian (Polynesian)” may have been categorized as “Other,” as well.

## Limitations

As the study design focused on adolescents, the sample size for adults was low and not representative of the adult population in New Caledonia. Moreover, we did not get precise information about the adults, such as age, SES or cultural community.

Although the participants formed a representative selection of adolescents attending school in New Caledonia in terms of the proportions of their socioeconomic characteristics, their numbers were relatively small. However, this limitation is mitigated by the objective nature of the data collected.

## Ethics Statement

The research met all legal requirements mandated by the Declaration of Helsinki. The protocol was approved by the Ethics Committee of New Caledonia (CCE 2018-06 001). All parents received an information letter and gave written informed consent prior to the adolescents’ participation by means of a note to be signed in the correspondence booklet, and the adolescents gave written informed consent to their own participation.

## CRediT authorship contribution statement

**Guillaume Wattelez:** Conceptualization, Methodology, Software, Formal analysis, Investigation, Data curation, Writing – original draft, Writing – review & editing, Visualization. **Stéphane Frayon:** Methodology, Investigation. **Olivier Galy:** Methodology, Investigation, Writing – review & editing, Supervision, Funding acquisition.

## Data Availability

ZenodoGENEActiv accelerometer files collected during the Eating cultures project (non-anonymized version - 1st part) (Original data). ZenodoGENEActiv accelerometer files collected during the Eating cultures project (non-anonymized version - 2nd part) (Original data). ZenodoGENEActiv accelerometer files collected during the Eating cultures project (non-anonymized version - 3rd part) (Original data). ZenodoInformation of participants involved in the Eating cultures project (non-anonymized information) (Original data). ZenodoGENEActiv accelerometer files collected during the Eating cultures project (anonymized version - 1st part) (Original data). ZenodoGENEActiv accelerometer files collected during the Eating cultures project (anonymized version - 2nd part) (Original data). ZenodoGENEActiv accelerometer files collected during the Eating cultures project (anonymized version - 3rd part) (Original data).
